# Design Principles and Applications of Ionic Liquids for Transdermal Drug Delivery

**DOI:** 10.1002/advs.202405983

**Published:** 2024-09-29

**Authors:** Sai Gao, Xueqing Cheng, Ming Zhang, Qiong Dai, Chaoyong Liu, Yunfeng Lu

**Affiliations:** ^1^ Beijing Advanced Innovation Center for Soft Matter Science and Engineering Beijing University of Chemical Technology Beijing 100029 P. R. China; ^2^ College of Life Science and Technology Beijing University of Chemical Technology Beijing 100029 P. R. China; ^3^ Department of Pathology Peking University International Hospital Beijing 102206 P. R. China

**Keywords:** ionic liquids, permeation enhancer, skin permeability, transdermal drug delivery

## Abstract

Ionic liquids (ILs) are salts with melting points typically <100 °C, composed of specific anions and cations. Recently, IL application has expanded into material engineering and biomedicine. Due to their unique properties, ILs have garnered significant interest in pharmacological research as solubilizers, transdermal absorption enhancers, antibacterial agents, and stabilizers of insoluble pharmaceutical active ingredients. The improvement of skin permeability by ILs is closely associated with their specific physicochemical characteristics, which are identified by their ionic composition. However, the existing literature on transdermal medication administration is insufficient in terms of a comprehensive knowledge base. This review provides a comprehensive assessment of the design principles involved in IL synthesis. Additionally, it discusses the methods utilized to assess skin permeability and provides a focused outline of IL application in transdermal drug administration.

## Graphical abstract

The physicochemical properties of ionic liquids (ILs) in transdermal drug delivery (TDD) directly affect drug permeation efficiency. Understanding the relationship between the molecular structure of ILs and their transdermal enhancement effect is essential for optimizing IL performance in transdermal drug administration. This article provides a comprehensive overview of the design principles, evaluation methods and applications for TDD.

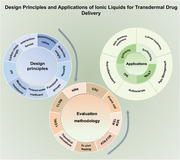

## 1 Introduction

The transdermal drug delivery system (TDDS) has gained increasing attention as a novel method for drug administration, with the advancement of drug delivery technology. TDD involves utilizing the skin as a drug administration pathway, enabling the drug to target local tissues or enter the bloodstream to exert its therapeutic effects.^[^ ^1^ ^]^ TDD provides several advantages, including avoiding the first‐pass effect, improving patient compliance, and providing flexibility and convenience in drug administration.^[^ ^2^ ^]^ This delivery method is particularly advantageous for drugs that are sensitive to hepatic first‐pass metabolism. Notably, transdermal patches containing drugs, such as fentanyl, nicotine, and nitroglycerin, have been successfully commercialized.^[^ ^3^ ^]^ However, TDDS faces challenges in its application, such as low skin penetration efficiency and limited drug delivery efficiency, which restrict the broader adoption of TDD for different drugs. Poor skin permeability of most drug molecules, especially macromolecular drugs, is a primary reason for these limitations. Additionally, skin irritation caused by organic solvents further restricts their application in TDD.

Numerous physical and chemical methods have been proposed to improve the skin permeability of drugs. The primary techniques used to facilitate drug penetration through the skin include ultrasound,^[^ ^4^ ^]^ microneedles,^[^ ^5^ ^]^ iontophoresis,^[^ ^6^ ^]^ liposomes,^[^ ^7^ ^]^ chemical permeation enhancers (CPEs),^[^ ^8^ ^]^ etc. Among them, CPEs have gained considerable attention due to their ease of application. CPEs reported in current literature involve polyethylene glycol, fatty acids, and aza‐type compounds,^[^ ^9^ ^]^ which improve drug transdermal efficiency by increasing drug solubility or altering skin tissue structure. However, these CPEs often exhibit irritating or sensitizing effects. In recent years, interest in exploring the use of a novel solvent type known as ionic liquid (ILs) in TDD has been growing. ILs are salts that are composed of asymmetric organic cations and organic or inorganic anions with melting points of <100 °C.^[^ ^10^ ^]^ ILs possess a range of unique properties, including excellent solvation capabilities, a wide liquid‐state temperature range, negligible vapor pressure, nonvolatility, and nonflammability, and are considered a green alternative to traditional organic solvents.^[^ ^11^ ^]^ ILs are categorized into three generations based on their composition. The first generation of ILs is characterized by toxicity and limited applications. Conversely, the second and third generations demonstrate improved biocompatibility and biodegradability, making them the current research focus.^[^ ^12^ ^]^

ILs demonstrate significant potential for improving the transdermal permeation of insoluble drugs due to their distinctive physicochemical properties.^[^ ^10^, ^13^ ^]^ As CPEs, ILs typically include amphiphilic or hydrophobic ions, which facilitate their penetration through the lipid layer of the skin. Currently, using ILs as CPEs has two primary approaches. One method involves applying ILs individually or in the form of (micro‐ or nano‐) emulsions to dissolve drugs that are challenging to solubilize as solvents or accelerators.^[^ ^14^ ^]^ The other approach involves synthesizing active pharmaceutical ingredients (APIs) into ILs to facilitate the transdermal delivery of the APIs.^[^ ^15^ ^]^ Studies have revealed that certain ILs significantly improve the transdermal efficiency of both small and large molecules, such as taurine, acyclovir, and insulin. The superior performance of ILs in improving transdermal permeation, compared to traditional chemical permeation enhancement methods, presents innovative opportunities for developing TDDS suited to an expanding spectrum of insoluble drugs.^[^ ^16^ ^]^ However, several factors limit the widespread application of ILs currently. Primarily, ILs possess inherent skin irritation, requiring the development of more biocompatible variants to mitigate this effect. Second, a notable limitation lies in the lack of standardized approaches for evaluating the synthesis and transdermal efficacy of ILs. Additionally, the suitability of drugs for administration with ILs is pivotal for their widespread adoption. Currently, consolidated literature that provides guidelines for designing ILs in TDD processes and methodologies for their transdermal evaluation is lacking. In this review, we focus on the rules and parameters of ILs that need to be considered during the design process. We investigated the intricate association between these parameters and the resultant transdermal efficiency. Additionally, we aim to comprehensively summarize various evaluation methods used to evaluate the efficacy of ILs in TDD. Furthermore, this work provides an overview of the application of frequently used ILs, including choline‐based and amino acid‐based ILs, for TDD. This study aimed to provide information that contributes to advancing TDD technology using ILs.

## 2 ILs: Pathways and Mechanisms for TDD

**Figure 1:**
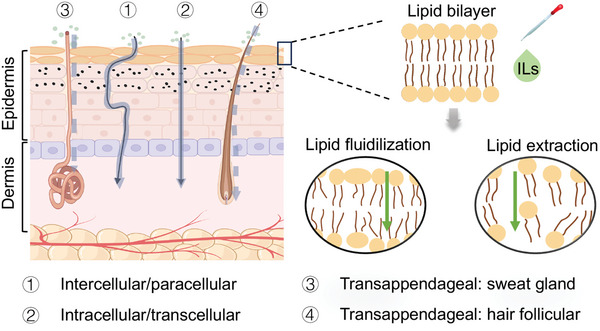
Skin‐permeation pathways and IL‐TDD mechanism.^[^ ^20^ ^]^ Created with BioRender.com.

In recent years, ILs have been widely utilized in TDDS, providing unique advantages over traditional methods. Understanding the mechanisms by which ILs facilitate TDD is crucial. Drug permeation through the skin primarily occurs via the skin's structure, specifically through two main pathways: 1)intercellular and 2) transcellular (**Figure** **1**). The transcellular pathway involves drugs passing through the SC cells to reach the viable epidermis. However, this pathway predominantly facilitates the delivery of hydrophilic compounds or small molecules due to the low permeability of SC cells and the requirement for drugs to undergo several hydrophilic/lipophilic distribution processes. In contrast, intercellular permeation depends on the lipid matrix within the interstitial spaces between cells for drug transport. Lipophilic drugs are predominantly absorbed into the viable epidermis via the intercellular pathway. Drug molecules are delivered through both pathways simultaneously in most cases.^[^ ^17^ ^]^ Additionally, other drug delivery routes involve the skin's appendages, including 3) sweat glands or 4) hair follicles.^[^ ^18^ ^]^ However, the small surface area available for absorption, accounting for only 0.1% of the total skin area, limits this pathway.^[^ ^19^ ^]^

Researchers have devised several strategies to enhance the efficiency of TDD. One promising approach involves using ILs, which improve the transdermal efficiency of drugs through multiple mechanisms (Figure 1). These mechanisms involve lipid fluidization, which increases lipid matrix fluidity and thereby improves permeability, and lipid extraction, which develops transient pores or pathways that facilitate drug diffusion. Additionally, ILs reduce barrier action and improve permeability by disrupting keratin in the SC. Various factors, including drug polarity, molecular weight, and other parameters, affect the ability of ILs to permeate the skin. **Table** **1** summarizes the mechanisms by which different ILs facilitate drug permeation through the skin, according to references. Noteworthily, further in‐depth studies are warranted to investigate additional mechanisms by which ILs facilitate TDD.

**Table 1:** Examples of ILs for TDD.

S. No	Drug	ILs	Mechanism	Refs.
1.	Dextran	[Choline] [Geranic Acid] (CAGE)	ILs induce lipid extraction, which replaces the skin lipids with ILs and water, thereby facilitating faster diffusion.	[21]
2.	NSAIDs (Ibuprofen∖Zaltoprofen∖Ketoprofen∖Naproxen∖Loxoprofen∖Diclofenac∖Flubiprofen∖Mefenamic acid)	[Ethylamine (EA)∖ Diethylamine (DEA)∖ Triethylamine (TEA)∖ Tripropylamine (TPA)∖ Triamylamine (TAA)] [NSAIDs]	The increased miscibility of drugs with the SC causes conformational disturbances and phase changes in the lipid bilayer.	[22]
3.	Ibuprofen	[Proline ethylester (ProOEt)] [Ibuprofen]	The [ProOEt] [Ibuprofen] compound primarily exists in its neutral form, facilitating its delivery across the hydrophobic SC layer.	[23]
4.	Peptides	[Choline] ([Cho] [FA])	The permeation mechanism entails peptides permeating through the intracellular lipids of the SC.	[24]
5.	Ketoprofen	[Piperine (PI)] [Ketoprofen (KP)] (KP‐PI)	An expected increase in permeability from ILs was anticipated for KP, as the favorable improvement in its lipophilicity promoted its partitioning into the SC and facilitated diffusion into the receptor compartment.	[25]
6.	Navitoclax (NAVI)	[Choline] [Octanoate] (COA)	The topical delivery of NAVI mediated by COA improved skin penetration and prolonged the drug's retention in the deeper skin layers for an extended period.	[26]
7.	Tretinoin	[Choline] [Tretinoin (Tr)]	The lipophilicity of active pharmaceutical ingredients ionic liquid (API‐IL) contributes to their high skin permeability.	[27]
8.	Dencichine	[1‐hydroxyethyl‐3‐methylimidazolium chloride] ([HOEIM] [Cl])/[1‐butyl‐3‐methylimidazolium dodecanesulfate] ([BMIM] [C_12_SO_3_])	The nanocarrier reduces the skin barrier properties by disrupting the regular and compact arrangements of corneocytes, thereby moderating the surface properties of the SC.	[28]
9.	/	[Oleic acid] [Propylene glycol (PG)]	The [oleic acid] [PG] system induced lipid extraction, which subsequently caused a reorganization of the SC structures.	[29]

## 3 Classification and Properties of ILs

**Figure 2:**
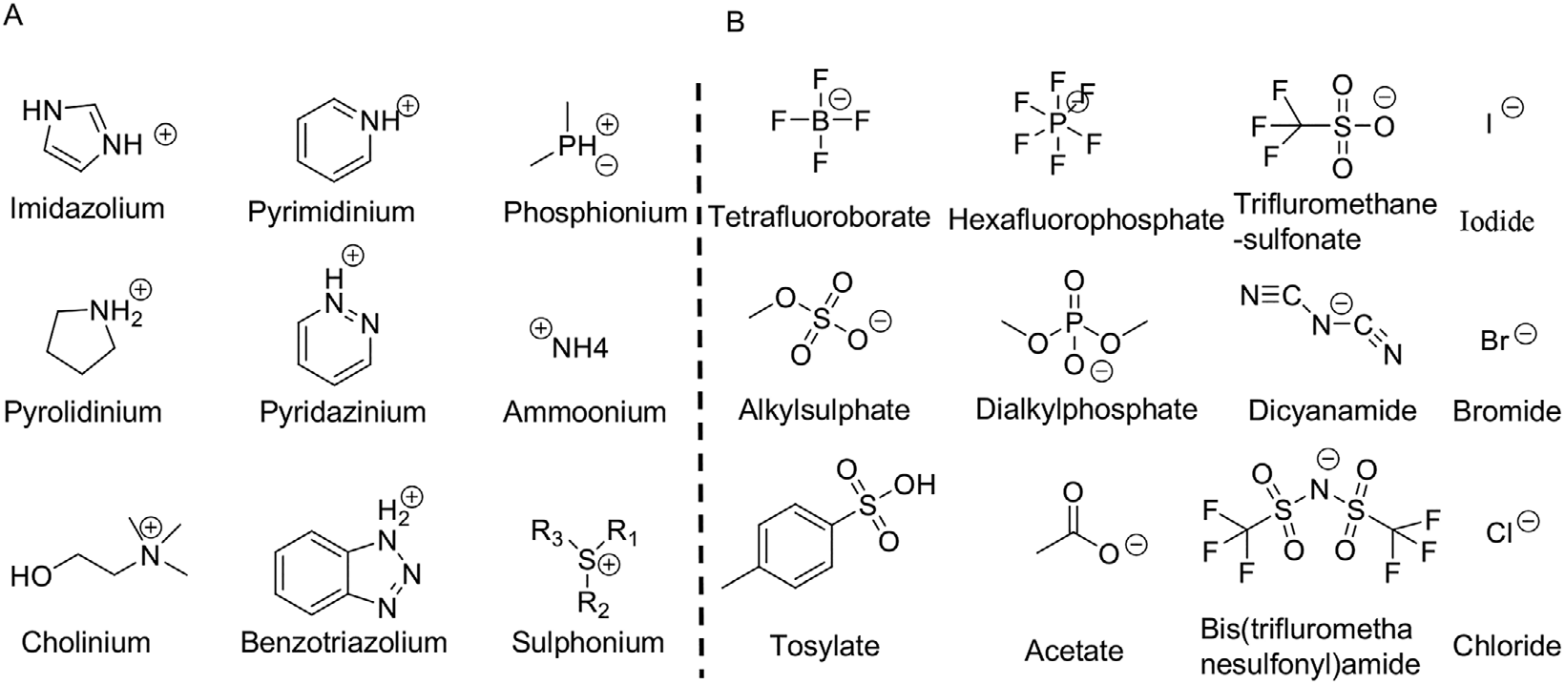
The structure of common cations A) and anions B) making up ILs.

Currently, three IL generations are available for application, with each generation distinguished by its unique chemical structures and properties. **Figure** **2** shows the structures of the common ILs. The first IL generation typically comprises bis‐basic imidazolium or alkyl pyridine cations paired with metal halide anions. These ILs demonstrate sensitivity to air and water, requiring processing and investigation under complete vacuum and inert atmosphere conditions,^[^ ^30^ ^]^ and are primarily used in electrochemical studies. The second generation typically features predominant cations, such as dialkyl imidazole, alkylpyridine, ammonium, and phosphorus, paired with anions, including halides, tetrafluoroborate, and hexafluorophosphate. ILs, composed of these cations and anions, demonstrate greater stability to air and water compared to those of the first generation. Consequently, they have revealed applications in physics and chemistry.^[^ ^31^ ^]^ Subsequently, the third IL generation was developed, using biocompatible ions, such as choline and amino acids, either individually or in combination with established active ions.^[^ ^12b^ ^]^ This recent development paves the way for their use in biomedical applications.

Previously, we emphasized that ILs have earned the moniker of “green solvents” due to their exceptional solvation properties, broad liquid‐state temperature range, negligible vapor pressure, nonvolatility, and nonflammability. These characteristics make them extensively used in developing TDDS. In this section, we will explore the remarkable properties of ILs, particularly focusing on the third IL generation.

First, ILs demonstrate a remarkable ability to remain in a liquid state over a wide range of temperatures, typically spanning from −90 to 300 °C or even broader. This unique characteristic allows their use under extreme temperature conditions, ensuring consistency in liquid form during routine application. This property facilitates convenient preservation and application. Importantly, this attribute is particularly advantageous in treating dermatological disorders, which frequently manifest over large surface areas and present difficulties for many TDD improvement strategies.^[^ ^32^ ^]^ The nonvolatility of ILs prevents drug volatilization during delivery, ensuring the precision of administered doses. Moreover, the polarity and amphiphilicity of ILs are important factors affecting their application. The polarity helps in drug solubilization in aqueous solutions. Meanwhile, amphiphilicity refers to the ability of some ILs to mix with both hydrophobic solvents and water. Amphiphilic ILs typically feature hydrophobic substituents at one end of the long chain and hydrophilic substituents at the other.^[^ ^33^ ^]^ Additionally, hydrophilicity is improved through hydrogen bonding to carboxyl groups, alcohol groups, and nitrogen atoms.^[^ ^34^ ^]^ The combination of these characteristics caused the widespread application of ILs in various biomedical fields. The liquid nature and miscibility of ILs in polar and nonpolar solvents play a crucial role in solubilizing hydrophobic drugs in aqueous solutions and overcoming hydrophobic barriers in the body. In particular, researchers have improved the dermal penetration of the hydrophilic macromolecule dextran with ILs composed of choline and malic acid. The dextran level delivered to the epidermis and dermis by the choline malate ([C] [M]) was approximately twice that of the dextran solution.^[^ ^35^ ^]^ ILs demonstrate low toxicity and irritation, rendering them biocompatible and suitable for use as carriers in TDD. The predominant composition of these ILs is either cationic, characterizing a choline group, or anionic, incorporating an amino acid group. Researchers have investigated the cytotoxicity of eight potential fatty acid‐based amino acid ILs. Remarkably, the findings indicated that all these fatty acid‐based amino acid ILs demonstrated a cell survival rate at least 10 times higher than conventional CPEs.^[^ ^36^ ^]^

## 4 Design Principles of ILs: Controllable Parameters

**Figure 3:**
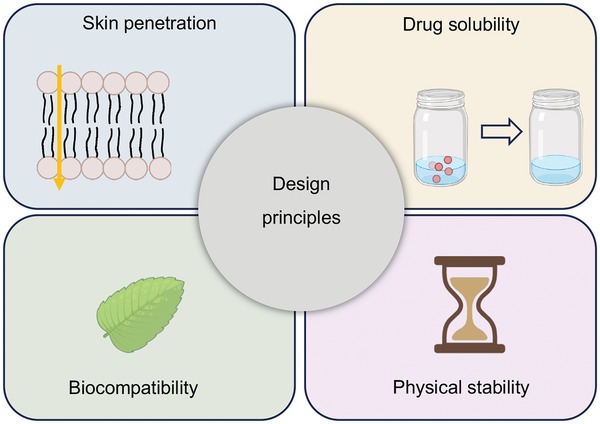
Design principles to be followed in TDD of ILs. Created with biorender.com.

The design principles of ILs for TDD involved drug solubility, skin penetration, biocompatibility, ILs, and physical stability (**Figure** **3**). First, ILs that significantly solubilize the drug must be selected to ensure adequate dissolution and retention within the IL medium. Second, ILs should facilitate drug penetration through the skin barrier by changing skin structure or interacting with skin components, thereby improving transdermal absorption. The selected ILs should exhibit good biocompatibility and low toxicity to ensure their safety for the skin and the human body. The properties of ILs, such as polarity, hydrophilicity/hydrophobicity, and ionic strength, can be adjusted by selecting appropriate cation and anion combinations, thereby affecting the drug's solubility and transdermal absorption. Finally, ensuring that ILs possess good physical stability and do not undergo phase separation or degradation during storage and use is crucial.

The anions and cations are carefully designed to optimize the properties and transdermal effects of ILs to achieve the aforementioned goals. Controllable parameters include cations and anions types and ratios, polarity, hydrophilicity/hydrophobicity, ionic strength, viscosity, electrical conductivity, and interactions with drugs and skin components. The solubility and transdermal absorption of the drug are effectively enhanced while ensuring biocompatibility and physical stability by optimizing these parameters. This optimization is grounded in extensive research on the mechanisms and applications of IL‐based TDD. The following is a summary of the controllable parameters of ILs and their optimal ranges (**Table** **2**).

**Table 2:** Controllable parameters and mechanisms of ILs design in TDD.

Parameters	Optimum value range	Mechanism	Refs.
Interaction forces between ions	2–6 kcal mol^−1^	The strong interaction between cations and anions increases the stability and solvation ability of ILs	[37]
Stoichiometric ratios of ions	Anion: Cation = 1: 2	Higher ion density and more uniform charge distribution will increase the solubility and transdermal effect of the drug	[38]
Carbon chain length of ions	C12–C18	Suitable lipophilicity and fluidity to penetrate through the skin	[39]
Molecular weight (M.W.) of ions	<500 Da	The smaller size, higher diffusion coefficient, and ability to penetrate the tight lipid bilayer structure of the skin more efficiently	[21, 40]
Molecular resistance and functional groups of ions	Long‐chain alkane	Increased steric hindrance reduces IL distribution into the SC. The increasing number of double bonds elevates the stereoscopic barrier. Functional groups containing hydrogen bond donors (such as hydroxyl groups) and acceptors (including carbonyl groups) improve the solubility and stability of drugs by forming hydrogen bonds with skin and drug molecules and promoting their transdermal absorption	[41]
Octanol–water partition coefficient	LogP: 1–5	Compounds with high LogP‐values (>5) may be too lipophilic, causing excessive accumulation in the skin lipid layer and difficulty in passing through water channels. Compounds with LogP‐values that are too low (<1) may be too hydrophilic to penetrate the lipid layer	[42]
Interaction forces between the loaded drug and ILs	Effective hydrogen bonding	The hydrogen bond interaction between the drug and the ILs improves drug solubility and stability, thereby enhancing the efficiency of TDD	[43]
Viscosity	Lower viscosity	The lower viscosity helps ILs flow and spread more easily, thereby increasing the efficiency of drug penetration through the skin	[44]
Heat stability	High thermal stability	ILs with good thermal stability can maintain their functional and structural integrity under these conditions, thereby ensuring effective drug release and absorption	[45]
pH value	5.5–7.0	The variable properties of SC proteins in an acidic environment improve skin permeability. Additionally, this pH range can reduce skin irritation	[46]

### 4.1 Interaction Forces Between Ions

Strong interactions between anions and cations in ILs identify the formation and stability of IL structure, which is crucial for ILs in TDDS.^[^ ^17b^ ^]^ For example, researchers used molecular dynamics simulations to demonstrate strong hydrogen bonding between choline and geranic acid using choline to geranic acid (CAGE) ILs.^[^ ^47^ ^]^ The primary interaction occurs between the carboxyl group of geranic acid and the hydroxyl group of choline. Tanner et al.^[^ ^17b^ ^]^ used 2D nuclear overhauser effect spectroscopy (NOESY) to investigate intraionic interactions. This technique uses nuclear magnetic resonance (NMR) data plotted on two frequency axes, leveraging diagonal peaks and crosstalk to unveil the spatial interactions between the underlying chemical groups (e.g., cations and anions) by quantifying the number of crosstalk occurrences. Another study revealed that NOESY data revealed that CAGE compositions containing a 1:2 ratio of choline to geranic acid caused the most significant transdermal penetration improvement.^[^ ^16b^ ^]^ Systematic anion modification demonstrated that ILs with weaker interionic interactions were most successful in transdermal transport. Furthermore, cationic modifications that further reduced interionic interactions also improved delivery efficacy.^[^ ^17b^ ^]^ Furthermore, Hong et al. investigated pyridine‐based ILs and revealed that strong hydrogen bonding and π–π interactions significantly improved the transdermal permeability of drugs.^[^ ^48^ ^]^

### 4.2 Stoichiometric Ratios of Ions

The cation‐to‐anion ratio is one of the most fundamental control variables in IL design, capable of significantly changing physical properties and interactions with biological tissues. Tanner et al.^[^ ^16b^ ^]^ used NMR hydrogen spectroscopy to monitor the average intra‐ and interion interactions across space within a CAGE. Further, they calculated the diffusion coefficients of molecules. The displacement of the hydroxyl proton on choline fluctuated with the cation‐to‐anion ratio. Additionally, the effect of different CAGE ratios on insulin permeation through the skin was investigated, revealing that a 1:2 ratio of choline to geranic acid facilitated insulin permeation. Tanner et al.^[^ ^17b^ ^]^ used CAGE to assess the effect of ionic stoichiometry on the skin permeation of a drug. The results revealed that the highest delivery efficiency was achieved with a molar choline‐to‐germanic acid ratio of 1:2. Other studies have revealed that choline and amino acid‐based ILs indicated that the transdermal permeability of the antiviral drug acyclovir was significantly improved by adjusting the cation‐to‐anion ratio.^[^ ^49^ ^]^ This improvement may be caused by changes in pH and other factors due to excess carboxylate, which improves the transdermal effect.

### 4.3 Carbon Chain Length of Ions

Chain length is correlated with hydrophilicity and lipophilicity, where short‐chain fatty acids tend to demonstrate reduced skin permeation due to their lack of lipophilicity. Conversely, excessively long alkyl chains hinder permeation because of their affinity for lipids in the SC, caused by hydrophobic interactions.^[^ ^41^ ^]^ Penetration improvement indicates that the mode of action of saturated fatty acids as enhancers depends on fatty acid penetration through the SC. Choi et al.^[^ ^50^ ^]^ investigated the effect of fatty acids on the in vitro transdermal penetration of donepezil base (DPB) and its hydrochloride salt (DPH), discovering that the relative in vitro rate of transdermal penetration of donepezil (DP) through the skin of hairless mice correlated with the length of the carbon chain of the fatty acid, following a parabolic association. The experiments investigating the transdermal facilitation effect of fatty acids on diclofenac by Kim et al.^[^ ^41^ ^]^ revealed a parabolic correlation between the enhancement effect and the length of the fatty acid carbon chain in saturated fatty acids ranging from C12 to C20 units. In contrast, they found that permeation increased with the carbon chain length for the monounsaturated fatty acid series, and oleic acid (C18:1) demonstrated the highest permeation enhancement effect. These distinct linear relationships indicate that the degree of carbon chain length influenced the transdermal effect.

### 4.4 Molecular Weight (M.W.) of ILs

Generally, the M.W. of a drug is a crucial factor affecting drug transdermal permeability. The skin readily permits the passage of small molecules, whereas larger molecules encounter greater challenges in traversing. The skin permeability of drug‐ILs demonstrated a notable parabolic association with their M.W. when the M.W. of the drug was substantial.^[^ ^22^ ^]^ Qi et al.^[^ ^21^ ^]^ investigated the effect of the M.W. of dextran, a macromolecular substance in CAGE (1:2), on its permeability through the skin. They revealed that in the presence of CAGE, the skin permeability of dextran with a medium M.W. (4, 20, 40, And 70 kDa) increased over 40‐fold compared to the control group with PBS. Another study revealed that ILs show significant potential in facilitating the transdermal delivery of high molecular weight (M.W) drugs, although this effect is more pronounced with smaller M.W. drugs.^[^ ^51^ ^]^ These studies demonstrate that this enhancement is limited as the M.W. increases, whereas ILs can significantly improve the transdermal delivery of drugs. Therefore, considering the M.W. of the drug molecules and performing comprehensive optimization in conjunction with the specific application to achieve the best transdermal delivery effect is important when designing and optimizing ILs for transdermal delivery systems.

### 4.5 Molecular Resistance and Functional Groups of Ions

The study revealed that the increased steric hindrance limited the partitioning of ILs into the SC lipid bilayer. Generally, the greater the molecular steric resistance, the lower the transdermal efficiency. Conversely, the smaller the molecular steric resistance, the easier the ILs penetrate the skin. Additionally, functional groups with certain lipophilicity (e.g., long‐chain alkyl, allyl) improve the skin permeability of ILs because these functional groups increase the affinity of ILs to the skin lipid layer. Kim et al.^[^ ^41^ ^]^ revealed that substituting the carboxylic acid portion of oleic acid with amide (oleamide) or hydroxyl (oleyl alcohol) groups diminished the transdermal effect. Theoretically, a corresponding penetration enhancement effect would be expected due to the low melting point of oleyl alcohol. However, oleic acid demonstrated the most potent penetration‐improving effect in this study, likely caused by the combined solubilization effect of the solvent PG and fatty acids. Additionally, increasing the number of double bonds elevates steric hindrance. The solubility of diclofenac decreases with the increase in double bonds, and the skin penetration rate demonstrates a parabolic relationship. This is because the kinks formed in the skin's lipid structure increase as the number of double bonds in unsaturated fatty acids elevates, thereby further improving drug penetration. However, the distribution of fatty acids into the SC lipid bilayer becomes limited as the number of double bonds continues to increase.

### 4.6 Octanol–Water Partition Coefficient

Lipophilicity, frequently expressed as the octanol–water partition coefficient, is a crucial property in drug action. It significantly affects pharmacokinetic and pharmacodynamic processes, as well as drug toxicity.^[^ ^52^ ^]^ The investigation of transdermal delivery of ILs revealed that the logarithm of the partition coefficient (logP) can be utilized to quantify the lipophilicity of the synthesized ILs. This helps identify their capacity to traverse the phospholipid bilayer of the skin. Florindo et al.^[^ ^53^ ^]^ used the octanol–water coefficient to characterize the partitioning properties, specifically the hydrophilicity, of ampicillin‐based ILs. Their results revealed that all ampicillin‐based ILs demonstrated higher logP‐values than ampicillin, indicating higher lipophilicity. In particular, ILs with stronger lipophilicity improved drug partitioning into the cell membrane. Octanol–water partition coefficients are typically determined using the shake flask method. Additionally, molecular dynamics simulations have been employed to calculate the predicted octanol–water partition coefficients of ILs.^[^ ^54^ ^]^

### 4.7 Interaction Forces Between the Loaded Drug and the ILs

ILs and drugs predominantly interact through hydrogen bonding. Hattori et al.^[^ ^43^ ^]^ used ^1^H NMR spectroscopy and observed variations in spectra at 6.1–6.5 ppm when altering the molar ratio of the drug nobiletin (NOB) to the CAGE. This observation indicates that NOB interacts with CAGE via hydrogen bonding interactions. Specifically, the hydrogen atom of the hydroxyl group in CAGE (6.1–6.5 ppm in the NMR spectra) serves as a hydrogen bond donor. Meanwhile, the oxygen atoms of the carbonyl group and methoxy group in NOB acted as multipoint hydrogen bond acceptors. Consequently, the hydrogen bonding between NOB and CAGE may contribute to improving the solubility of NOB. Moreover, CAGE may improve the solubility of flavonoids in addition to NOB, considering that all flavonoids contain at least one oxygen atom in their backbone structure. Zhang et al.^[^ ^55^ ^]^ synthesized an actarit‐triethylamine‐ketoprofen IL and revealed that the semi‐ionic hydrogen bonding between the IL and the APIs plays a crucial role in improving drug solubility and permeability. This bonding mechanism aids in the formation of a stable and effective drug delivery system, significantly improving the therapeutic potential of the APIs.

The transdermal efficiency of drugs usually cannot be determined by a single factor mentioned above. It is the synergistic effect of multiple factors. Factors, such as the characteristics of the delivered drugs and the design of the TDDS, exhibited an important effect on the transdermal efficiency of drug delivery besides the chemical properties of the ILs themselves. Therefore, optimizing the composition of ILs following the specific application and comprehensively considering various factors to develop an IL‐based TDDS with high transdermal efficiency and stable drug delivery is necessary.

## 5 Recommended Framework for Standardized Evaluation of ILs for TDD

Adopting a structured approach that not only focuses on the theoretical design but also incorporates rigorous evaluation and development strategies is essential to effectively develop TDDS using ILs. In this section, we will first outline a recommended framework for the standardized assessment of ILs, which is crucial in verifying their transdermal efficiency after parameter modulation. This framework will be further detailed in the upcoming sections, where we will investigate specific assessment methods and discuss the development strategies required to ensure both the efficacy and safety of IL‐based TDDS.

### 5.1 Evaluation Methods for IL‐Based TDDS

In this section, we will focus on summarizing standardized evaluation methods utilized to confirm the transdermal efficiency of ILs following parameter modulation. Additionally, we will discuss the advantages and limitations of each method. Moreover, we will investigate the use of these methods to rationally design ILs to optimize their effectiveness in TDD (**Table** **3**).

**Table 3:** Evaluation methods for transdermal efficacy and mechanisms in IL drug delivery.

Category	Evaluation Method	Principle	Advantages	Limitations	Refs.
Transdermal Efficacy	Franz diffusion cell	In vitro skin‐permeation testing	Quantitative analysis and standardized method	In vitro results may not align with in vivo	[56]
	Confocal laser scanning microscopy (CLSM)	Fluorescence imaging of drug penetration depth	Visualization of penetration depth and quantitative and qualitative analyses	Fluorescence labeling requirement and limited resolution	[18b]
	*Ex vivo* skin retention	Drug concentration detection in excised skin layers	Drug retention assessment in skin tissues	Tissue sampling requirement	[56]
	In vivo permeation	Drug concentration measurement in blood/tissue	Systemic availability evaluation of the drug	In vivo experiment requirement	[57]
Transdermal mechanism of action	ATR–FTIR Spectroscopy	Infrared spectroscopy to detect lipid changes in the skin	Nondestructiveness and microstructural change detection	Unavailable information on deeper layers	[58]
	NMR spectroscopy	Chemical shift to detect hydrogen bonding interactions	Molecular‐level information	Complex equipment and sample preparation	[59]
	Transepidermal water loss (TEWL)	Water evaporation measurements and reflection of skin barrier function	Noninvasiveness and ease of measurement	Environmental factor influences	[18b]
	Differential scanning calorimetry (DSC)	Heat absorption and release measurement and skin phase transition detection	Sensitiveness, suitability for studying skin phase transitions	Limited to thermodynamic properties	[60]
	Electron microscope	microstructural alteration visualization in the skin SC	High‐resolution imaging and suitability for detailed microstructure analysis	Complex sample preparation and surface or ultrathin layer observation limitation	[60]

We achieved the following goals through systematic evaluation. First, we can accurately quantify the degree of effects of different parameters on transdermal efficiency. Second, these methods help us determine key parameters and potential synergistic effects on transdermal efficiency. Furthermore, we optimize the composition and structure of the ILs through these evaluations to maximize their ability to facilitate transdermal transmission. Finally, these assessments provide a solid scientific basis and guidance for IL application in TDDS.

### 5.2 Development Strategy for IL‐Based TDDS

A structured approach is crucial in developing TDDS using ILs to ensure both efficacy and safety, particularly when targeting specific diseases. The first step in this process involves a detailed analysis of the drug's characteristics, including its solubility, M.W., and stability. These properties must be considered in the context of the drug's therapeutic application, such as pain management or chronic disease treatment, to identify its suitability for transdermal delivery. Understanding these characteristics is critical, as they affect appropriate IL selection that can improve the drug's permeability through the skin while maintaining stability and effectiveness in the targeted treatment area.

After understanding the drug's properties, suitable ILs that complement these characteristics and enhance transdermal delivery are selected. This involves choosing the appropriate cation and anion combinations to optimize solubility and skin permeability while ensuring that the ILs are biocompatible and appropriate for the specific therapeutic application. In particular, the IL must facilitate the controlled release of the drug at the site of application in conditions requiring localized treatment, such as topical pain relief. Thus, IL selection is a crucial step in tailoring the TDDS to the specific requirements of the disease being treated.

After selecting ILs, the formulation is synthesized, emphasizing optimizing the IL properties to suit the disease context. This includes considerations, such as the desired drug release rate and the specific site of application. The formulation is then subjected to in vitro testing, where it is assessed using techniques, such as Franz diffusion cells, to evaluate skin permeation and drug retention in skin tissues. These tests are crucial in identifying the initial effectiveness of the IL‐based TDDS and how it may perform in a clinical setting, particularly in terms of its potential effect on the targeted disease.

The next phase involves in vivo testing, where the formulation is evaluated for systemic drug absorption, skin irritation potential, and overall biocompatibility in relevant animal models. These tests are developed to mimic the clinical conditions under which the TDDS would be utilized, providing valuable data on its safety and efficacy. The results from both in vitro and in vivo testing are then used to optimize IL composition, ensuring that the formulation achieves the therapeutic goals of the disease treatment while minimizing side effects.

Once optimized, the formulation is prepared for clinical trials, with all required documentation compiled for regulatory approval. This stage ensures that the TDDS meets all safety and efficacy requirements for its intended therapeutic application. The product can be commercialized, focusing on scaling up production and ensuring that the formulation meets the requirements of the target market, after obtaining regulatory approval. Postmarket surveillance is crucial to monitor the long‐term safety and efficacy of the TDDS, with adjustments made as necessary according to clinical outcomes and patient feedback. This structured approach ensures that IL‐based TDDS is not only effective but also safe and specified for the disease being treated.

## 6 IL Application in TDD

ILs, recognized as “green solvents,” are intriguing chemicals with a wide range of applications in various research areas, such as topical drug delivery and TDD. They are used as solvents/solubilizers or surfactants, as well as in API‐IL formation and transdermal vaccine formulations for cancer treatment,^[^ ^61^ ^]^ antimicrobials for soft tissue infections,^[^ ^62^ ^]^ cosmetics,^[^ ^63^ ^]^ and dermatological treatments.^[^ ^64^ ^]^ Summarizing the various disease treatment applications where ILs have exhibited great potential is crucial, considering their unique advantages and the growing interest in their use (**Table** **4**). Our focus primarily revolves around the third IL generation, specifically the application of biocompatible ILs in TDD, for which a concise summary is provided.

**Table 4:** IL application in disease treatment.

Application	ILs	API	Refs.
Pain management and anti‐inflammatory	Ibuprofen proline ethylester ILs	Ibuprofen, naproxen, hydrocortisone, and dexamethasone	[65]
Local anesthesia	Ketoprofen‐based ILs	Benzocaine, procaine, and tetracaine	[66]
Anticancer	Oleic acid‐based ILs	5‐Fluorouracil	[67]
Antibacterial and antiviral	Choline Carboxylic Acids ILs	Mannitol and acyclovir	[68]
Treatment of skin diseases	CAGE	siRNA	[69]
Hyperglycemic	Tributyl(tetradecyl)phosphonium ILs and CAGE	Gliclazide and insulin	[70]
Vaccine	Choline‐fatty acids ILs	Antigen peptide	[24]

### 6.1 ILs as Solvents

The use of ILs as solvents represents one of their most traditional applications, largely due to the unique properties inherent in these compounds. These properties improve drug solubility, skin permeability, and stability, thereby providing promising avenues for addressing various diseases.

**Figure 4:**
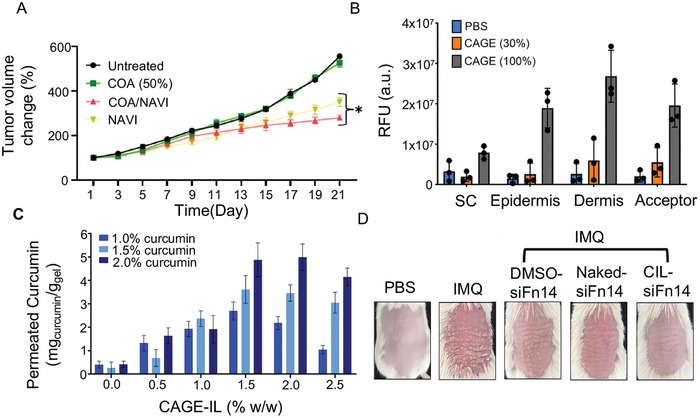
A) Tumor volume measured during the treatment.^[^ ^26^ ^]^ Copyright 2022, Elsevier. B) Quantitative results of nanoparticles observed in *ex vivo* porcine skin layers utilizing Franz diffusion cell.^[^ ^73^ ^]^ Copyright 2022, John Wiley and Sons. C) Net amounts of curcumin permeated from the gel formulations containing various concentrations of curcumin and CAGE.^[^ ^71^ ^]^ Copyright 2022, Elsevier. D) Representative graphs of psoriasis‐like skin lesions on day 7.^[^ ^72^ ^]^ Copyright 2024, Elsevier.

TDDS plays a crucial role in both preventing and treating cancer and cancer‐like illnesses. In 2022, Huda et al.^[^ ^26^ ^]^ formulated a topical preparation that comprises navitoclax (NAVI), a BCL‐2 inhibitor known for inducing apoptosis, along with [choline] [octanoate] IL (COA), aimed at treating early‐stage melanoma. The researchers revealed that the topical application of NAVI facilitated by COA improved skin penetration and prolonged drug retention within deeper skin layers. Moreover, the topical administration of NAVI exhibited superior efficacy in killing cancer cells compared to its oral counterpart (**Figure** **4**A). These results indicate that the topical approach could provide a safe and effective option for improving the clinical management of skin cancer.

ILs play a crucial role in stabilizing proteins. Bekdemir et al.^[^ ^73^ ^]^ integrated ILs with nanosensors to detect thrombus formation promptly. A nanodiagnostic agent designed for thrombogenesis detection, which is an effective delivery to subdermal blood vessels, was achieved using CAGE (Figure 4B). This integration notably improves the stability of polyethylene glycol (PEG) nanosensors. Researchers revealed that recombinant thrombin displayed robust activity against peptide substrates when incorporated into nanosensors within a 30% CAGE formulation through in vitro fluorescence assays.

Moreover, ILs have garnered extensive use in treating dermatological conditions. Boscariol et al.^[^ ^71^ ^]^investigated the efficacy of CAGE as a permeation enhancer for curcumin, and their results indicated that CAGE significantly improves the transdermal permeation of curcumin (Figure 4C). This discovery holds significant promise for treating various skin conditions, including, but not limited to, psoriasis.

Li et al.^[^ ^72^ ^]^ developed a composite IL (CIL) to facilitate the transdermal delivery of Fn14 siRNA (siFn14) to keratinocytes to regulate psoriasis‐associated inflammatory responses. Their results demonstrated that CIL‐siFn14 effectively suppressed keratinocyte proliferation and inflammation (Figure 4D). The therapeutic potential of siRNA extends to various skin diseases. Dharamdasani et al.^[^ ^64b^ ^]^ used ILs, which are known for their capacity to noncovalently complex with siRNA and efficiently deliver it topically. Their study, supported by in vitro and in vivo experiments, revealed the effective delivery of siRNA to the skin through a combination of complementary and synergistic strategies involving ILs, causing significant inhibition of relevant gene expression in mice. However, further investigation is warranted to assess its clinical toxicity.

ILs are effective in antimicrobial applications by facilitating the delivery of antibiotics. Zakrewsky et al.^[^ ^68b^ ^]^ achieved improved penetration of cefadroxil, an antibiotic, into deeper tissue with the assistance of CAGE. A wound model of biofilm infection, demonstrating bacterial death of >95% after a 2 h treatment, confirmed the in vivo efficacy of CAGE. This study establishes the use of ILs for augmenting topical drug delivery and antibiotic efficacy.

As solvents, ILs primarily improve the solubility of drugs through their strong interactions with drug molecules. This increased solubility subsequently improves the drug's penetration through the skin. Many drugs that are poorly soluble in water are typically administered orally as solid tablets, frequently causing low absorption rates and significant side effects. By contrast, ILs address these issues by improving drug solubility and facilitating transdermal delivery, thereby providing a promising alternative for administering such drugs with better absorption and reduced adverse effects.

### 6.2 ILs Made up of API

Beyond their role as a solvent for improving the solubility of insoluble drugs, ILs also enhance drug solubility by forming API‐IL complexes. This involves incorporating the active drug ingredient into the IL structure, thereby effectively making the drug an integral component of the resultant ILs.^[^ ^70^ ^]^ Such formulations facilitate improved skin penetration, thereby improving therapeutic outcomes.

**Figure 5:**
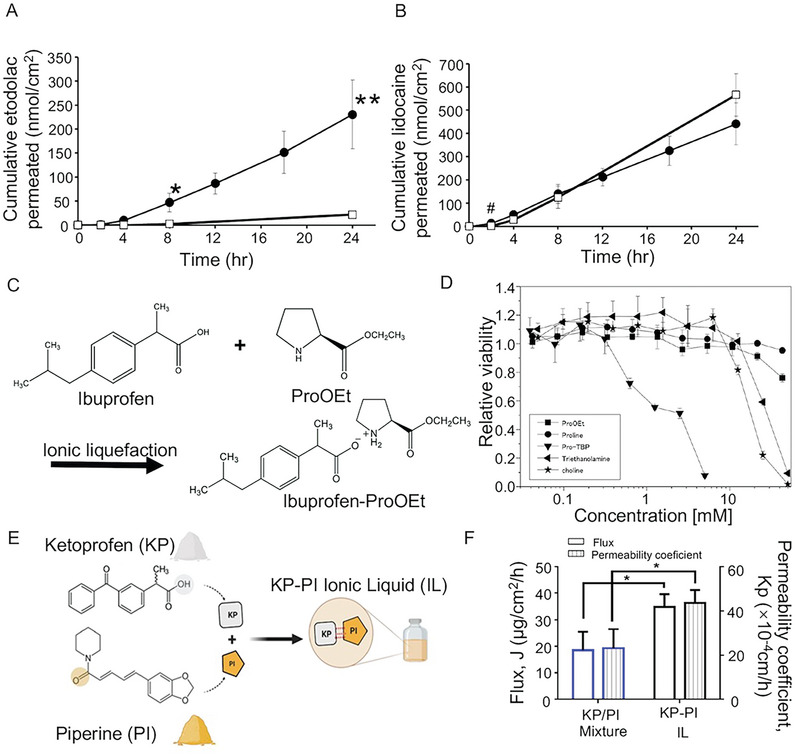
A) and B) Skin‐permeation profiles for etodolac in Etoreat (●) and E‐alone (□), as well as lidocaine in etoreat (●) and L‐alone (□).^[^ ^74^ ^]^ Copyright 2022, Elsevier. C) The synthetic scheme of proline ethyl ester‐ibuprofenate (ibuprofen‐ProOEt).^[^ ^23^ ^]^ Copyright 2016, Portion. D) Cytotoxicity of various types of cations, ProOEt, proline (Pro), Pro‐TBP, triethanolamine, and choline.^[^ ^23^ ^]^ Copyright 2016, Portion. E) Diagram illustrating chemical structures of KP and PI, as well as IL formation.^[^ ^25^ ^]^ Copyright 2022, Elsevier. F) Flux and permeability coefficient (Kp) of KP/PI mixture and KP‐PI IL through rat skin.^[^ ^25^ ^]^ Copyright 2022, Elsevier.

Nonsteroidal anti‐inflammatory drugs (NSAIDs) are extensively used in treating various inflammatory conditions. Miwa et al.^[^ ^74^ ^]^ addressed the challenge of the poor water solubility of etodolac, an NSAID, by preparing ILs from it to improve its hydrophobicity and skin permeability. The researchers utilized etodolac and lidocaine (in a 1:1 molar ratio) as raw materials. In vitro skin‐permeation tests revealed that etodolac patches significantly improved the skin permeation of etodolac by 9.3‐fold compared to etodolac alone. Moreover, lidocaine enhanced the skin permeation of etodolac by sacrificially converting it into ILs (**Figure** **5**A,B). Furukawa et al.^[^ ^23^ ^]^ introduced amino acid esters (AAEs) as effective and biocompatible counteracting ions for formulating API‐IL. IL formulations were achieved by pairing equimolar amounts of the NSAID ibuprofen as an anion with AAEs as a cation (Figure 5C). The results revealed that AAEs, serving as counterbalancing cations, demonstrated substantially lower cytotoxicity than conventional cations (Figure 5D). This indicates the considerable potential of AAE in developing novel IL drug formulations. In contrast, Sara A. Hassan et al. synthesized an IL form of a novel anti‐inflammatory drug, KP.^[^ ^25b^ ^]^ KP was paired with PI in this IL, and an equimolar KP‐PI IL was developed through solvent evaporation (Figure 5E). This IL formulation enabled transdermal administration and significantly improved both the solubility and skin permeability of KP (Figure 5F), causing more pronounced efficacy.

**Figure 6:**
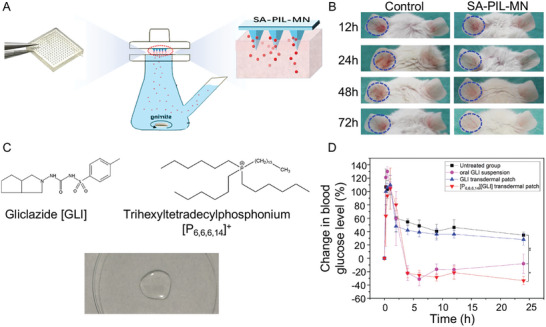
A) Picture of the prepared SA‐PIL‐MN patches.^[^ ^64^ ^]^ Copyright 2020, Elsevier. B) Schematic representation of the skin acne therapy in a mouse model via SA‐PIL‐MN administration.^[^ ^64^ ^]^ Copyright 2020, Elsevier. C) Chemical structure and appearance of ILs.^[^ ^1^ ^]^ Copyright 2021, Elsevier. D) Changes in blood glucose levels after oral administration and transdermal delivery (*n* = 3).^[^ ^1^ ^]^ Copyright 2021, Elsevier.

Zhang et al.^[^ ^64a^ ^]^ pioneered the development of a salicylic acid (SA)‐containing API poly IL (API PIL)‐based microneedle (MN) patch, as depicted in **Figure** **6**A. These innovative API PIL‐based microneedles demonstrated promising capabilities for improving acne treatment (Figure 6B), indicating potential applications in addressing various skin disorders.

Zhou et al.^[^ ^1a^ ^]^ initially transformed the second‐generation sulfonylurea gliclazide (GLI) into an IL that contains tributyl (tetradecyl) phosphine (Figure 6C) and formulated it into a transdermal patch. This patch demonstrated notable transdermal efficacy. Importantly, comparative analysis revealed that the transdermal patch generated superior hypoglycemic effects compared to oral suspension under both fasting and feeding conditions (Figure 6D), confirming the feasibility of achieving systemic effects through topical IL administration.

API‐IL complex formation introduces a strategic design principle for improving drug solubility and permeability. This strategy involves several key design parameters: lowering the melting point, increasing solubility and viscosity, and improving fluidity. These factors collectively enhance the drug's synergistic effects and skin permeability. Furthermore, the ability to integrate multiple active ingredients into a single IL complex not only simplifies the formulation but also improves overall therapeutic efficacy. This approach holds crucial potential in treating various diseases, thereby providing a versatile and effective method for improving drug delivery and therapeutic outcomes.

### 6.3 ILs as Surfactants

ILs primarily help in formulating drug carriers, such as microemulsions and nanoparticles. Drugs that are insoluble in water, most organic solvents, or only slightly soluble in water, can be effectively delivered transdermally using these carriers by incorporating ILs. This approach not only facilitates transdermal delivery but also improves delivery efficiency.^[^ ^75^ ^]^

**Figure 7:**
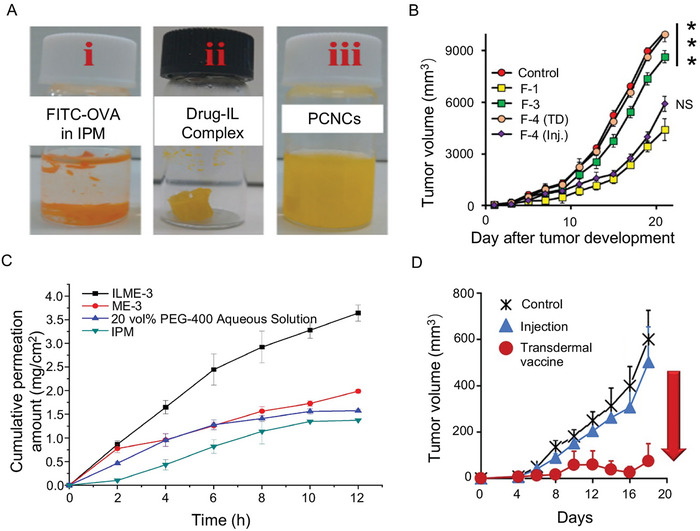
A) Transdermal process of PCNCs.^[^ ^76^ ^]^ Copyright 2022, American Chemical Society. B) Tumor growth inhibition.^[^ ^76^ ^]^ Copyright 2022, American Chemical Society. C) Permeation kinetic curves of Ars across different systems.^[^ ^80^ ^]^ Copyright 2020, Elsevier. D) Effect of transcutaneous cancer vaccination via the IL/EtOH/IPM system.^[^ ^24^ ^]^ Copyright 2020, American Chemical Society.

Uddin et al.^[^ ^76^ ^]^ utilized ILs served as surfactants to encapsulate ovalbumin (OVA) proteins, which form a protein‐containing nanocarrier (PCNC) (**Figure** **7**A). This PCNC demonstrated significantly improved transdermal distribution and delivery, exhibiting 25‐ and 28‐fold higher efficacy than its aqueous counterpart. The results indicated markedly elevated transdermal distribution and delivery of PCNC, causing a substantial tumor growth inhibition (Figure 7B). Toyofuku et al.^[^ ^77^ ^]^ achieved notable antitumor effects by delivering antisense oligonucleotides (ASOs) using ILs and solid‐in‐oil (S/O) dispersion technology. Their results revealed that lipid‐based IL surfactants, characterized by high biocompatibility and skin permeability, effectively facilitated the transdermal penetration of ASOs. Moreover, ASO encapsulation enabled their intracellular delivery. Cholate oleate [Cho]^[^ ^78^ ^]^ is recognized as a surface‐active IL (SAIL), which forms micelle nanoconjugate formulations (MFs) when coloaded with sorbitan monolaurate (Span‐20). Both in vivo and in vitro skin stimulation experiments revealed that these MFs based on SAIL [Cho]^[^ ^78^ ^]^ demonstrated their potential as biocompatible nanocarriers. Additionally, they demonstrated the capability to load the chemotherapeutic drug paclitaxel (PTX) for tumor therapy.^[^ ^61b^ ^]^

Islam et al.^[^ ^79^ ^]^ developed oil‐encapsulated IL microemulsions (MEFs) for the transdermal delivery of insulin. Their efficacy and stability have been relatively robust. Zhang et al.^[^ ^80^ ^]^ introduced a microemulsion system that incorporates an IL and a deep eutectic compound to improve the transdermal absorption of artemisinin (Ars). Their study revealed that the formulated microemulsion effectively promotes the transdermal delivery of Ars. Moreover, it is anticipated to be a carrier for facilitating the transdermal delivery of various other hydrophobic natural drugs. Zhang et al.^[^ ^80^ ^]^ integrated ILs with microemulsions to augment the transdermal penetration of Ars drugs, thereby fabricating API‐IL‐based microemulsions. In vitro transdermal assays demonstrated a substantial enhancement in Ars transport across the skin, with a permeation flux within 6 h that was both three times higher than that of the myristic acid isopropyl ester and isopropyl myristate systems (Figure 7C). Shu et al.^[^ ^81^ ^]^ developed thermo‐responsive hydrogels that incorporate IL microemulsions (IL‐ME) for transdermal delivery of methotrexate (MTX). These microemulsions were established using water, Tween 20, and CAGE. The solubility of MTX in IL‐ME was nine‐fold higher compared to phosphate buffer solution, demonstrating significant improvement. Moreover, the penetration effect was notably improved, as indicated by histopathological sections of mouse skin in the treatment group (Figure 7D).

The use of ILs as surfactants is congruent with the design principle of improving drug delivery systems by enhancing solubility and permeability. This approach enables effective drug carrier formulation, thereby optimizing drug delivery and therapeutic outcomes. The ability of ILs to change surface tension and produce stable microemulsions or nanoparticles, which encapsulate drugs and improve their transdermal penetration, is key. The success of IL‐based nanocarriers in transdermal applications emphasizes the potential of this strategy in developing advanced drug delivery systems.

### 6.4 ILs for Enhanced Transdermal Vaccines

Transdermal vaccination stands out as an advanced and patient‐friendly immunization approach due to immune cell abundance in the skin and its administration simplicity. However, achieving this effect requires the use of CPES, similar to those described in drug delivery methods discussed earlier. Researchers have investigated the use of ILs for vaccine delivery to improve drug delivery and stabilize formulations.

Tahara et al.^[^ ^24^ ^]^ utilized [Cho] [FA] as biocompatible ILs which facilitated the solubilization of water‐soluble antigenic peptides within an oil‐based skin penetration enhancer. Remarkably, the transdermal delivery flux of peptides increased 28‐fold when using IL [Cho] [C18:1] in conjunction with an oil‐based penetration enhancer, compared to delivery via an aqueous carrier. Moreover, IL‐mediated transdermal vaccination effectively suppressed tumor growth in vivo compared to injection. Peptide dissolution in oil‐based skin penetration enhancers mediated by biocompatible ILs presents a promising strategy for TDD.

IL application in transdermal vaccines emphasizes their potential to improve the efficacy and stability of vaccine formulations. Stabilizing protein structures and promoting transdermal penetration are crucial for effective vaccine delivery. The successful use of ILs in transdermal vaccination emphasizes the importance of selecting appropriate ILs to achieve efficient and effective immunization strategies.

## 7 Conclusions and Outlook

This work provides a comprehensive review of the key parameters and indicators crucial for TDDS design and implementation, thereby leveraging the advantages of ILs. It involves pertinent design principles, permeability evaluation methodologies, and a focused summary of IL application in TDD. Specifically, it highlights the use of biocompatible ILs in topical drug delivery, emphasizing their efficacy in treating dermatological disorders and cancer. The promising potential of ILs in TDD indicates that their application will continue to expand in the future.

We determined the significant challenge in the screening and optimization of CPEs for TDD due to the interrelated nature of their physicochemical parameters by summarizing the previous research. Literature that provides a definitive formula or rule to elucidate these associations is lacking. This deficiency causes uncertainty and a lack of direction in screening CPEs for optimizing TDD formulations. The unclear design principles governing ILs may have caused this ambiguity. Therefore, proposing a novel improved action parameter to assess the physicochemical parameters of CPEs comprehensively becomes imperative. This approach aims to address the above‐mentioned difficulties qualitatively and quantitatively. Additionally, ILs can be customized to address specific drug delivery challenges. This involves selecting and optimizing IL components according to the physicochemical properties required for particular drugs and therapeutic applications. Customizability enables the fine‐tuning of ILs to achieve the desired balance between solubility, permeability, and stability, just as nanoantidotes are tailored to target specific toxins.^[^ ^82^ ^]^

Furthermore, the clinical application of ILs holds great promise, but it is currently limited due to concerns about their toxicity and a lack of clarity regarding their transdermal permeation mechanism. We advocate for further research to elucidate the transdermal mechanisms of ILs, thereby leveraging computational simulation technologies to overcome these limitations. Additionally, efforts should be directed toward developing biocompatible ILs for a broader range of diseases, thereby expanding their clinical utility.

The physicochemical properties of ILs in their application in the field of transdermal drug delivery are directly associated with the transdermal efficiency of drugs. Therefore, an in‐depth summary and understanding of the constitutive association of ILs, i.e., the correlation between the molecular structure of ILs and their transdermal improvement effect, is crucial for optimizing and enhancing the performance of ILs in transdermal drug delivery applications.

## Conflict of Interest

The authors declare no conflict of interest.
